# Ameliorative action of “daitongxiao” against hyperuricemia includes the “uric acid transporter group”

**DOI:** 10.3389/fphar.2024.1300131

**Published:** 2024-06-25

**Authors:** Lijie Zheng, Yuanmei Bai, Yan Wan, Feifan Liu, Yuhuan Xie, Jinglin He, Peixin Guo

**Affiliations:** ^1^ College of Ethnic Medicine, Yunnan University of Chinese Medicine, Yunnan, Kunming, China; ^2^ College of Basic Medical Sciences, Yunnan University of Chinese Medicine, Yunnan, Kunming, China

**Keywords:** daitongxiao, hyperuricemia, uric acid transporter group, pharmacodynamics, mechanisms

## Abstract

This study aimed to investigate the potential mechanisms involved in the therapeutic effects of daitongxiao (DTX) on hyperuricemia (HUA). DTX was administered to two animal models of HUA via gavage feeding: HUA quail model (a uricotelic animal with urate oxidase deficiency), treated continuously for 35 days post-HUA induction, and HUA rats (an animal with active urate oxidase), treated continuously for 28 days post-HUA induction. HUA was induced in quail by administering a solution of sterile dry yeast powder via gavage feeding, while in rats, it was induced by intragastric gavage feeding of a solution of adenine and ethambutol hydrochloride. DTX improved overall health; increased bodyweight; reduced renal index, serum urate levels, serum xanthine oxidase activity, blood urea nitrogen, and creatinine; and enhanced urinary and fecal uric acid (UA) excretion in these two animal models. The results of hematoxylin and eosin and hexamine silver staining of kidney sections revealed that DTX significantly mitigated HUA-induced renal structural damage and inflammatory response. The results of quantitative real-time polymerase chain reaction, Western blotting, and immunofluorescence analyses revealed that DTX downregulated the renal expression levels of glucose transporter 9 (GLUT9) and upregulated the renal expression levels of organic anion transporters (OAT1 and OAT3) in both HUA models. Thus, the findings of this study suggest that DTX suppresses the progression of HUA by modulating the expression of the UA transporter group members.

## Graphical Abstract

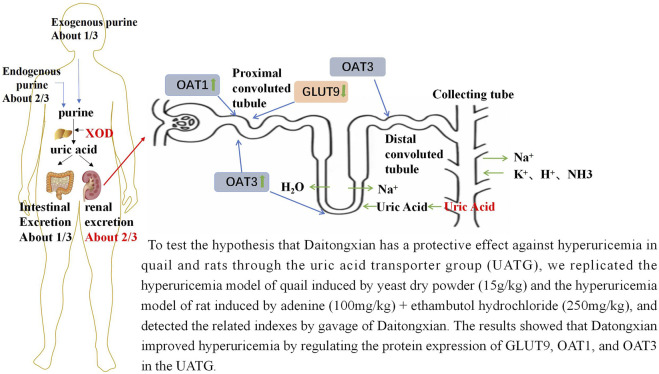

## Highlights

• Daitongxiao (DTX) has the potential to exert therapeutic effects for ameliorating hyperuricemia (HUA) in HUA quail and rat models.
• DTX ameliorates the damage in renal functions in the HUA quail model, induced by the solution of yeast dry powder, and in the HUA rat model, induced by intragastric administration of the solution of adenine and ethambutol hydrochloride.
• DTX exerted anti-hyperuricemic effects by decreasing reabsorption of urate in renal tubules, increasing uric acid (UA) secretion in renal tubules, and promoting UA excretion through regulation of GLUT9, OAT1, and OAT3 of the uric acid transporter group (UATG).

## 1 Introduction

Hyperuricemia (HUA), a metabolic disease, is caused by dysregulation of purine metabolism and is characterized by upregulated uric acid (UA) levels (Yanai et al., 2021). HUA is diagnosed in both men and women based on the criteria that fasting blood urate levels are >420 μmol/L on 2 separate days when patients are on a regular purine diet (Becker et al., 2005). Recent changes in dietary structure have increased the annual prevalence of HUA, especially in younger individuals. The global rate of HUA prevalence, which continues to increase annually, is 0.86%–2.20% (0.83%–1.98% in men and 0.07%–0.72% in women) (Liu et al., 2015). Currently, effective treatments for HUA are not available. Most patients with HUA require long-term or lifelong treatment with UA-lowering drugs. Drugs used in the clinical treatment of HUA are mainly categorized into two groups, uricostatic drugs and uricosuric agents. The uricostatic drug allopurinol suppresses the synthesis of UA by inhibiting xanthine oxidase (XOD) (Carnovale et al., 2014). However, long-term allopurinol use increases the risk of hepatotoxicity (Park et al., 2019; Fontana et al., 2021). Propofol decreases the serum urate (SU) levels by inhibiting the renal tubular reabsorption of UA and consequently downregulates SU levels (Masuda A et al., 1997; Haring et al., 2013). Probenecid is associated with severe intestinal adverse reactions (Horikawa et al., 2002; Choi JG et al., 2018). Rasburicase, a recombinant UA oxidase (de Bont JM et al., 2015), promotes oxidation of UA into allantoin, which is subsequently excreted through urine (Patel et al., 2017). Higher serum urate levels are reported to have a poor outcome on vascular event rates (Weir et al., 2003) and direct association with cardiovascular disease (CVD) events in hypertensive patients (Alderman et al., 1999). It was also shown that among urate-lowering treatments (ULTs), ULTs with allopurinol, febuxostat, or rasburicase in gout patients had almost no significant effect in CVD events (Zhang and Pope, 2017). However, rasburicase increases the risk of developing CVDs. Traditional ethnomedicines, especially natural medicines, have unique advantages in treating HUA (Sakti et al., 2020).

Daitongxiao (DTX) has been used by the Dai people in Yunnan Province, China, for the treatment of “Longmengshahou” (HUA in modern medicine) for more than 2,500 years (Zheng et al., 2022). The use of DTX to alleviate diseases caused due to elevated UA levels has been documented in the 21st century Dai medical undergraduate education book *Clinical Science of Dai Medicine* (Wu et al., 2022; Zheng et al., 2022). Clinical studies have shown that out of 29 HUA patients treated with DTX (20 g/day/person, with a duration of 14 days), 25 patients had improved serum urate (SU) levels with an overall efficacy rate of 86.22%. DTX comprises *Elsholtzia rugulosa* Hemsl (Lamiaceae; Herba Elsholtzia) and *Pinus tabuliformis* Carrière (Pinaceae; Pini lignum nodi) at a ratio of 3:1. Modern studies have demonstrated that the main active metabolites of the whole plant of *E. rugulosa* are triterpenes, flavonoids, sterols, glycosides, as well as volatile oil components such as dehydrogenated Elsholtzia, Elsholtzia, and thymol. It has antipyretic, detoxifying, antioxidant, and UA-lowering effects (Liu F et al., 2024). Moreover, the nodules and branching nodes of *P. tabuliformis* are used as therapeutics and comprise flavonoids and volatile oils as the main active metabolites (Liu F et al., 2024), such as α-pinene, which has anti-inflammatory and antioxidant effects (Liu F et al., 2024).

Experimental studies have reported that DTX can significantly downregulate serum UA, blood urea nitrogen (BUN), and creatinine (Cre) levels in the yeast powder-induced HUA mouse model (Zheng et al., 2022), exerting renal protective effects. Previous studies have demonstrated that in the adenine + ethambutol hydrochloride-induced HUA rat model, DTX significantly increased urinary excretion of UA and decreased SU levels (Liu F et al., 2024). Thus, we hypothesized that the anti-hyperuricemic mechanism of DTX may involve modulation of the expression of UA transporter group (UATG) members, resulting in reduction in tubular reabsorption of UA or upregulation of tubular secretion of UA or both in renal tubules.

Currently, multiple members of the UATG, which involve urate reabsorption transporters (primarily located in the renal tubule) and secretion transporters (located in the renal tubule and intestine), are cloned and well-characterized (Mandal and Mount, 2015; Mandal et al., 2017). The human urate reabsorption group of transporters includes URAT1 (encoded by *SLC22A12*), GLUT9 (encoded by *SLC2A9*), OAT10 (encoded by *SLC22A13*), and OAT4 (encoded by *SLC22A11*). The urate secretion group of transporters includes ABCG2 (encoded by *ABCG2*), MRP4/ABCC4 (encoded by *ABCC4*), OAT1 (encoded by *SLC22A6*), OAT3 (encoded by *SLC22A8*), NPT1 (encoded by *SLC17A1*), and NPT4 (encoded by *SLC17A3*). These transporters of the UATG are suggested to be critical for maintaining urate homeostasis and are potential targets for future drug development for treatment of HUA (Mandal and Mount, 2015; Mandal et al., 2017). Approximately 99% of SU is freely filtered through the glomerulus, and approximately 90% of the filtered urate is reabsorbed in the kidney tubules, while <10% is excreted. Hence, the UATG is proposed to be involved in maintaining SU homeostasis.

In the urate reabsorption pathway, urate from the luminal glomerular filtrate is reabsorbed into the proximal tubular epithelial cells via apical URAT1, OAT10, and OAT4. The reabsorbed urate is transported from the proximal tubular epithelial cells into the blood via GLUT9, a sole membrane potential-driven urate transporter located in the basolateral membrane of the proximal tubular epithelium. In the urate secretion pathway, urate from the blood first enters the renal tubular epithelial cells via the basolateral urate transporters OAT1 and OAT3 and is transported from the proximal tubular epithelium into the renal tubular lumen via apical secretion transporters ABCG2, ABCC4, NPT1, and NPT4 (Mandal and Mount, 2015; Mandal et al., 2017). As UA exists as an anion at physiological pH (pKa of UA = approximately 5.8), it requires transporters/ion channels to cross the biological membrane.

The mechanism underlying the anti-gouty arthritis activity of DTX has been previously reported (Liu F et al., 2024). However, the anti-HUA activity of DTX is unknown. In this study, DTX has been demonstrated to exert anti-HUA activities through modulation of the expression of urate reabsorption and secretion transporters in the quail [in which UA metabolism is similar to that in humans because of the deficiency of urate oxidase/uricase that converts UA to allantoin (Wu et al., 2023)] and rat (in which urate oxidase is active) models of HUA, as well as exerts protective effects on renal tissues.

## 2 Materials

### 2.1 Experimental animals

Male Dyfak quails aged 40 days (bodyweight: 130 ± 10 g) were purchased from Beijing Huafukang Biotechnology Co., Ltd (Animal License No.: SCXK [Beijing] 2019-0008). Sprague–Dawley (SD) rats (n = 70) of specific pathogen-free grade aged 8 weeks (bodyweight 200 ± 20 g) were purchased from Beijing Sibeifu Biotechnology Co., Ltd (Animal License No.: SYXK [Dian] K2018-0005). The animals were maintained under the following conditions: temperature, 23°C ± 2°C; humidity, 50% ± 5%; and access to food and water, *ad libitum*. Animal care and experiments were performed based on the guidelines of the Animal Welfare and Management Act and the Guide for the Care and Use of Laboratory Animals of the National Research Council of the United States of America. Animal experiments were reviewed and approved by the Ethics Committee of the Yunnan University of Chinese Medicine (Animal Ethical Approval No.: R-062021167). All experimental protocols conformed to the guidelines of the Declaration of Helsinki and the Ethical Standards of the World Medical Association.

### 2.2 Drugs

DTX, which consists of *E. rugulosa* and *P*. *tabuliformis* at a ratio of 3:1, was purchased from Dai Medical Hospital, Xishuangbanna, Yunnan, China, and authenticated by Prof. Deqiang Feng, an expert in botanical drug identification from Yunnan University of Traditional Chinese Medicine. This study used benzbromarone and Tongfengshu capsules (TFS capsules) as positive control drugs to promote UA excretion. Benzbromarone, a commonly used anti-hyperuricemic drug, inhibits reabsorption of UA in renal tubules, decreasing the blood concentration of UA and exerting therapeutic effects on HUA (Xue et al., 2023). The TFS capsule, a Chinese medicinal preparation, clears heat, removes toxins and dampness, and functions as a diuretic. Thus, the TFS capsule is suitable for treating HUA caused by dampness–heat stasis (Yang and Ding, 2013). This study comparatively analyzed the effects of DTX and TFS capsule on HUA and renal UA excretion. The following materials were purchased from different vendors: benzbromarone (Lundgermann Pharmaceutical Co., Ltd; Lot No. J20180056), TFS capsules (Shaanxi Panlong Pharmaceutical Group Co., Ltd; Lot No. 20220503), yeast powder (Solarbio Co., Ltd; Lot No. Y8020), adenine (McLean Co., Ltd; Lot No. C11998881), and ethambutol hydrochloride (Picasso Co. Ltd; Lot No. K4NXVO1S-25g).

### 2.3 Quality control and main chemical composition of DTX

All the botanical components constituting DTX were purchased from Xishuangbanna Dai Hospital in Yunnan Province. The contents of the active metabolites complied with the requirements of the Chinese Pharmacopoeia and local laws and regulations (Table 1). Additionally, a previously established method was used for extraction of the botanical drugs, which ensured the stability and reproducibility of the pharmacological effects of metabolites (Table 1). This study determined the percent composition of the main chemical metabolites of botanical drugs in DTX (see Supplementary Table S1). The main active metabolites of *E. rugulosa* were monoterpenes, sesquiterpenes, and flavonoids, which exert antipyretic, detoxifying, antioxidant, and UA-lowering effects (Zheng et al., 2022). Moreover, the main active metabolites of *P. tabuliformis* were sesquiterpenes, terpene alcohol fractions, and flavonoids, such as α-pinene, which exert anti-inflammatory, UA-lowering, and renal protective effects (Zheng et al., 2022).

**TABLE 1:** Quality standards of DTX.

Drug name	Active metabolites	Control standard
Herba *Elsholtzia rugulosa*	Ethanol leachate	13.0% minimum
	Essential oil	0.3% minimum
Pini lignum nodi	α-Pinene	0.1% minimum
	Ethanol leachate	22.0% minimum
	Essential oil	0.4% minimum

### 2.4 Reagents

The kits for the estimation of UA (Cat. #C009-2-1), XOD (Cat. #C010-2-1), BUN (Cat. #A001-3), and Cre (Cat. #A003-1-2) were purchased from Nanjing Jiancheng Bioengineering Co., Ltd, China. Goat anti-rabbit IgG-488 (Cat. #SA00013-2) and goat anti-mouse IgG-594 (Cat. #SA00013-3) antibodies were purchased from Three Eagles Biotechnology Co., Ltd., China. Anti-OAT3 (Cat. #AF5749) and anti-OAT1 (Cat. #DF8582) antibodies were purchased from Affinity Co., Ltd., China. The anti-GLUT9 antibody (Cat. #67530-1-Ig) was purchased from Proteintech Co., Ltd., China. The anti-GAPDH antibody (Cat. #GR217575-73) was purchased from Abcam, Waltham, United States. Anti-Rabbit IgG (Cat. #7074s) was purchased from Cell Signaling Technology Co., Ltd., MA, United States.

### 2.5 Equipment

A Multiskan™ FC Microplate Photometer (Cat. #1410101) was purchased from Thermo Fisher Scientific Co., Ltd., China. A low-temperature high-speed centrifuge (Cat. #H1-16KR) was purchased from Hunan Kecheng Instrument Co., Ltd., China.

A fully automatic digital pathology slide scanner (Cat. #KF-PRO-005-EX) was purchased from Ningbo Jiangfeng Biological Information Technology Co., Ltd., China. An ultrapure water analyzer (Arium Bagtank 50) was purchased from Ningbo Dansbottom Environmental Protection Technology Co., Ltd., China.

## 3 Methods

### 3.1 Drug dosage

DTX human dosage to animal dosage conversion (Liu F et al., 2024): the clinical human dosage of DTX is 20 g of raw drug/day/person. Based on the principle of body surface area conversion, the clinically equivalent dose of DTX (1.8 g/kg bodyweight) was set as the low dose for quails and rats. The medium dose was two times the low dose (3.6 g/kg bodyweight), while the high dose was four times the low dose (7.2 g/kg bodyweight). Following previous studies, TFS capsules and benzbromarone (Yang and Ding, 2013) were administered at doses of 0.259 and 20 mg/kg bodyweight, respectively.

### 3.2 Drug preparation

Following the established method, 900 g of *E. rugulosa* and 300 g of *P. tabuliformis* were mixed at a ratio of 3:1, ground into coarse powder, and evenly mixed. The mixture was then soaked in 12 times the amount (by weight) of water for 0.5 h in a flask. The aqueous extract of DTX was prepared three times in 1.5 h. The three aqueous extracts of DTX were mixed, filtered through a gauze, and freeze-dried to form an infusion. The DTX infusion was diluted with distilled water to concentrations of 0.72, 0.36, and 0.18 g/mL to prepare DTX of doses 7.2, 3.6, and 1.8 g/kg bodyweight, respectively.

Yeast dry powder solution preparation: Following a published protocol (Ma et al., 2024), yeast dry powder was administered at a dose of 15 g/kg bodyweight. The yeast dry powder (15 g) was first autoclaved, dissolved in 10 mL of distilled water, and configured as a 1.5 g/mL suspension. The suspension was ultrasonicated for 10 min and stored in a refrigerator at 4°C. Before each gavage, the suspension was shaken well.

Adenine and ethambutol hydrochloride solution preparation: Adenine (100 mg) and ethambutol hydrochloride (250 mg) were autoclaved, dissolved in 10 mL of distilled water, and configured into appropriate concentrations for gavage administration in animals, except those in the control group. The animals (rats) were gavaged with adenine (100 mg/kg bodyweight) + ethambutol hydrochloride (250 mg/kg bodyweight) in the morning once a day for 28 days.

Preparation of TFS capsule solution: TFS capsules (0.259 g) were dissolved in 10 mL of double-distilled water to prepare a 0.0259 g/mL solution.

Preparation of benzbromarone solution: Benzbromarone powder (20 mg) was weighed using a precision electronic balance and dissolved in 10 mL of double-distilled water to prepare a 2 mg/mL benzbromarone suspension, which was stored at 4°C. The suspension was mixed well before use each time.

### 3.3 Treatment strategy for the HUA quail model with DTX (modeling, grouping, and drug administration)

**FIGURE 1:**
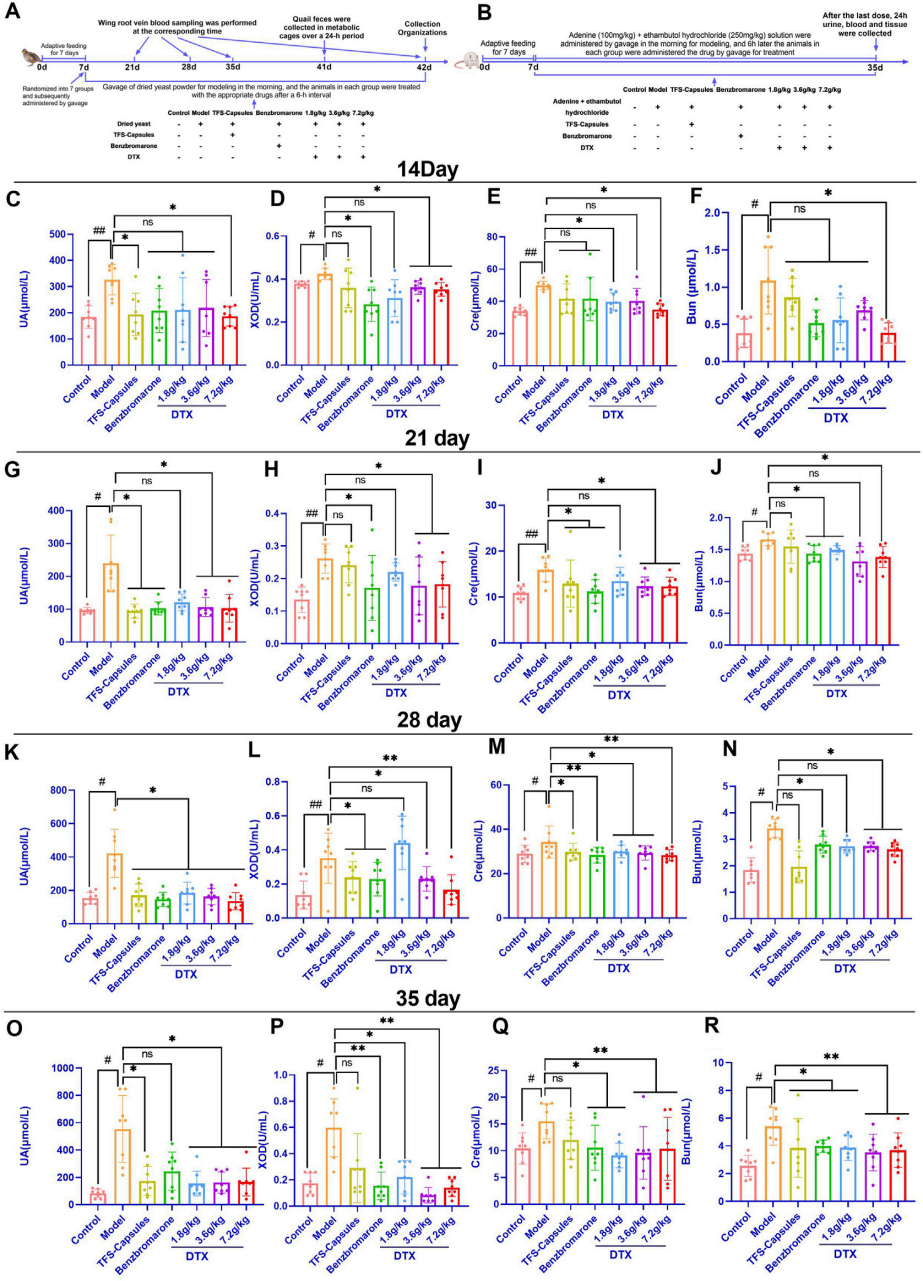
Daitongxiao (DTX) downregulates uric acid (UA) levels and xanthine oxidase (XOD) activity and improves renal function in quail with renal injury caused by hyperuricemia (HUA) (n = 8). **(A)** Timeline of establishing the quail HUA model and treating with TFS capsules, benzbromarone, or DTX. **(B)** Timeline of establishing the rat HUA model and treating with TFS capsules, benzbromarone, or DTX. **(C)** Quail serum UA level on day 14. **(D)** Quail serum XOD activity on day 14. **(E)** Quail serum creatinine (Cre) level on day 14. **(F)** Quail serum blood urea nitrogen (BUN) level on day 14. **(G)** Quail serum UA level on day 21. **(H)** Quail serum XOD activity on day 21. **(I)** Quail serum Cre level on day 21. **(J)** Quail serum BUN level on day 21. **(K)** Quail serum UA level on day 28. **(L)** Quail serum XOD activity on day 28. **(M)** Quail serum Cre level on day 28. **(N)** Quail serum BUN level on day 28. **(O)** Quail serum UA level on day 35. **(P)** Quail serum XOD activity on day 35. **(Q)** Quail serum Cre level on day 35. **(R)** Quail serum BUN level on day 35. ^#^ *p* < 0.05 and ^##^ *p* < 0.01 compared with the control group. ^*^ *p* < 0.05 and ^**^ *p* < 0.01 compared with the model group; ns, *p* > 0.05.

Healthy male quails (n = 56) were allowed to acclimatize for 1 week before the experiment and randomly divided into seven groups for treatment based on bodyweight (n = 8 quails/group): control, model, benzbromarone (20 mg/kg bodyweight), TFS capsule (0.259 g/kg bodyweight), low-dose DTX (1.8 g/kg bodyweight), medium-dose DTX (3.6 g/kg bodyweight), and high-dose DTX (7.2 g/kg bodyweight) groups. Animals in the test/model groups, but not those in the control group, were gavaged with sterile dry yeast powder (15 g/kg bodyweight) suspension in the morning for 35 consecutive days. After 6 h of daily gavage of the sterile dry yeast powder suspension (15 g/kg bodyweight), the animals in the treatment groups were gavaged with benzbromarone, TFS capsules, or DTX. The quails in the control and model groups were gavaged with an equal amount of distilled water (10 mL/kg bodyweight) once a day for 35 days (Figure 1A).

### 3.4 Evaluation of quail health and blood collection

The gross health, mental state, morphology, feces, and activity of quails in different groups were monitored. A comprehensive evaluation of quail health was performed.

Collection of serum samples from experimental quails for determination of biochemical indicators of HUA (UA, XOD, Cre, and BUN content)

Blood samples were collected from the wing root vein on days 14, 21, 28, and 35 post-gavage (the blood collection time for each quail was 1–2 min, and the blood volume was 0.8–1 mL), left undisturbed for 30 min, and centrifuged at 4°C and 1,503 *g* for 10 min. The UA, XOD, Cre, and BUN levels in the supernatant were determined using the kits, following the manufacturer’s instructions.

### 3.5 Collection of fecal samples from experimental quails for determination of UA content

On day 34 post-gavage, the quail fecal samples were collected from the metabolic cage for 24 h. The fecal samples (1 g) were washed with 2 mL saline and centrifuged at 4°C and 1,503 *g* for 10 min. The UA content in the supernatant was analyzed using a kit, following the manufacturer’s instructions.

### 3.6 Determination of the renal index of experimental quails

Quails in different groups were anesthetized with 3% sodium pentobarbital (120 mg/kg bodyweight) on day 35 post-gavage. The kidneys were carefully pulled out using ophthalmic forceps after opening the abdominal cavity. Excess fascia and fat were removed and weighed. The left kidney of each quail was used for pathological analysis, while the right kidney of each quail was stored in a freezer at −80°C. The following formula was used to calculate the renal index:

$$\text{Kidney index}=\frac{\text{Bilateral}\;\text{kidney}\;\text{weight}\;\left(g\right)}{\text{Quail}\;\text{body}\;\text{weight}\;\left(g\right)}\times 100\%.$$

### 3.7 Hematoxylin and eosin staining of kidney sections of the HUA quail for histopathological examination

The left kidney of each quail was fixed with 4% paraformaldehyde solution for 24 h and subjected to paraffin sectioning, dewaxing, and dehydration. The sections were subjected to hematoxylin and eosin (HE) and hexamine Ag staining. Renal histopathological analysis was performed using a fully automated digital pathology section scanner to evaluate renal inflammatory cell infiltration, glomerular structure, tubular epithelial cells, renal fibrosis, and protein tubular pattern.

### 3.8 DTX treatment regimen for the HUA rat model (modeling, grouping, and drug administration)

Healthy adult SD rats (n = 70) were allowed to acclimatize for 1 week before the experiment and randomly divided into seven groups (10 rats/group): control, model, TFS capsules, benzbromarone, low-dose DTX (1.8 g/kg bodyweight), medium-dose DTX (3.6 g/kg bodyweight), and high-dose DTX (7.2 g/kg bodyweight) groups.

Rats in all groups, except those in the control group, were gavaged with adenine (100 mg/kg bodyweight) + ethambutol hydrochloride (250 mg/kg bodyweight) in the morning for 35 consecutive days. After 6 h of daily gavage of the adenine (100 mg/kg bodyweight) + ethambutol hydrochloride (250 mg/kg bodyweight) suspension, the animals in different groups were gavaged with specific doses of TFS capsules, benzbromarone, or DTX. Rats in the control and model groups were gavaged with the same volume of distilled water (10 mL/kg bodyweight) once a day for 28 days (Figure 1B).

### 3.9 Evaluation of the general health of the HUA rat model

The gross health, mental state, morphology, feces, and activities of rats in different groups were examined. A comprehensive evaluation of rat health was performed.

### 3.10 Collection of urine samples to measure UA and Cre levels in the HUA rat model

After administering the last dose of DTX, the 24-h urine samples were collected in a rat metabolic cage. The urinary contents of UA and Cre were analyzed using the appropriate kit, following the manufacturer’s instructions.

### 3.11 Collection of serum samples to measure the UA, XOD, Cre, and BUN levels in the rats

At 24 h post-administration of the last dose of drugs, rats in different groups were anesthetized through intraperitoneal injection of 1% sodium pentobarbital (40 mg/kg bodyweight). Blood samples were collected from the abdominal aorta of rats (4–5 mL blood/rat for 30–45 s) and centrifuged at 1,503 *g* and 4°C for 10 min to separate the serum. The levels of UA, XOD, Cre, and BUN in the serum were analyzed using the kits, following the manufacturer’s instructions.

### 3.12 Calculation of fractional urinary UA excretion in rats

The levels of 24-h UA excretion and UA excretion fraction were calculated according to the following formula:

$$24h\text{ UA excretion}=24\text{ hurine volume }\left(\text{mL}\right)\times \text{UUA content }\left(\mu \text{mol}/L\right),$$

$$\text{UA excretion fraction}=\frac{\text{UUA}\;\text{content}\;\left(\mu \text{mol}/L\right)\times \text{Serum}\;\text{Cre}\;\text{level}\;\left(\mu \text{mol}/L\right)}{\text{Blood}\;\text{UA}\;\text{levels}\;\left(\mu \text{mol}/L\right)\times \text{Urinary}\;\text{Cre}\;\text{level}\;\left(\mu \text{mol}/L\right)}\times 100\%.$$

### 3.13 Evaluation of the morphology of rat kidneys

Rats from different groups were anesthetized, and the kidneys were removed and fixed. Before fixing, the appearance and morphology of kidneys, including the color and texture, were recorded.

### 3.14 Calculation of the renal index of rats

The bodyweight and total weight of bilateral kidneys were recorded. The left kidney of each rat was used for pathological analysis, while the right kidney was stored in a −80°C freezer for further studies. The renal index was calculated as follows:

$$\text{Rat kidney index}=\frac{\text{Rat}\;\text{bilateral}\;\text{kidney}\;\text{weight}\;\left(g\right)}{\text{Rat}\;\text{bodyweight}\;\left(g\right)}\times 100\%.$$

### 3.15 Slide preparation for histopathological analyses of rat kidneys

The rat kidneys were fixed with 4% paraformaldehyde solution for 24 h and subjected to paraffin sectioning, dewaxing, dehydration, and HE staining. The renal histopathological analysis was performed using a fully automatic digital pathology section scanner to evaluate renal inflammatory cell infiltration, glomerular structure, and tubular epithelial cells.

### 3.16 RNA extraction from quail kidneys and quantitative real-time polymerase chain reaction

Quail kidneys (60 mg) stored at −80°C were accurately weighed and lysed in 500 μL of lysis solution. The tissues were homogenized using an electric high-speed homogenizer. The total RNA was extracted using an RNA isolation kit. The RNA purity was determined using a SmartSpec Plus nucleic acid and protein detector. The isolated RNA from kidney samples was reverse-transcribed using a PrimeScript RT Reagent kit with a gDNA Eraser (perfect real-time) and a quantitative real-time polymerase chain reaction (qRT-PCR) instrument. qRT-PCR analysis was performed under the following conditions: pre-denaturation at 95°C for 3 min, followed by 40 cycles of denaturation (at 94°C for 10 s), annealing (at 55°C for 30 s), and extension (at 72°C for 2 min). A primer set specific for β-actin mRNA was used as the internal reference standard. The relative gene expression levels were determined using the 2^−ΔΔCT^ method. The designed intron-spanning primer sets were synthesized by Shanghai Shenggong Bioengineering Co., Ltd. The primer sequences are shown in Table 2.

**TABLE 2:** Sequence of quail kidney gene primers.

Gene name	Upper/lower primer	Sequence
GLUT9	Forward primer	5′-TGC​TGT​ACG​ATG​GCA​AGT​CA-3′
	Reverse primer	5′-CAA​GGG​TCT​CAA​CAG​CAC​CA-3′
OAT1	Forward primer	5′-CTG​CGC​CTA​CAT​CTT​CAC​CG-3′
	Reverse primer	5′-CCA​CGT​CCT​CCA​CAG​TTT​CG-3′
OAT3	Forward primer	5′-TCG​CCT​ACG​CCG​TCC​CAC​A-3′
	Reverse primer	5′-TTC​CTT​CCC​CGC​CAG​CAC​C-3′
β-Actin	Forward primer	5′-GAT​GAA​GCC​CAG​AGC​AAA​AGA-3′
	Reverse primer	5′-ACC​AGA​GGC​ATA​CAG​GGA​CAG-3′

### 3.17 Immunoblotting analysis of quail kidney homogenates

Quail kidneys were weighed, and the lysates of the quail kidneys were prepared using radioimmunoprecipitation assay (RIPA) lysis buffer containing phenylmethylsulfonyl fluoride (PMSF) (RIPA buffer:PMSF = 100:1; kidney weight:lysis buffer = 1:10). The protein concentration in the lysate was determined using a bicinchoninic acid kit. The lysate and 5× sampling buffer were mixed (4:1) such that the total protein concentration of each sample was 5 μg/μL. The samples were boiled at 95°C for 5 min, placed on ice for rapid cooling, aliquoted, and stored at −80°C. Next, the samples (15 μL/lane) were subjected to constant-voltage electrophoresis (180 V) for 40 min. The resolved proteins were electroblotted (100 V, 80 min) onto a polyvinylidene membrane. The membrane was blocked with 5% milk in Tris-buffered saline containing Tween-20 (TBST, pH 8.0) at room temperature for 1 h. Then, the membrane was probed with the anti-GLUT9 (1:1,000), anti-OAT1 (1:2,000), anti-OAT3 (1:1,000), and anti-GAPDH (1:1,000) primary antibodies (diluted in TBST buffer, pH 8.0) at 4°C overnight, followed by washing thrice with TBST and incubating with the rabbit anti-rat secondary antibodies (1:5,000) at room temperature for 1 h. After washing five times with TBST, the blots were incubated with an enhanced chemiluminescence (ECL) solution for 4–5 min. To perform quantitative Western blotting, the relative intensity of the GLUT9, OAT1, and OAT3 bands was measured after normalization (the ratio of the intensity of the target protein bands to that of the internal reference housekeeping protein GAPDH).

### 3.18 Immunofluorescence analysis of quail kidney sections

The quail kidney sections on glass slides were dewaxed in xylene, dehydrated by washing in absolute ethanol, and washed in water for 3–5 min. The sections were then incubated with anti-GLUT9 (1:500), anti-OAT1 (1:200), or anti-OAT3 (1:200) antibodies for 1 h, followed by rinsing thrice with phosphate-buffered saline containing Tween-20 (PBS-T). Next, the sections were incubated with a FITC-conjugated secondary antibody (1:400) for 1 h. The sections were then mounted with mounting medium (Cat. #80312-379 3181, Jiangsu Sitai Experimental Equipment Co., Ltd., China) containing the fluorescent dye 4′,6-diamidino-2-phenylindole (DAPI) (Cat. #C0065, Solarbio Co., Ltd., China) for nuclear staining. The fluorescence signals of GLUT9, OAT1, or OAT3 were detected using a fluorescence microscope (Cat. #XSP-C204; CIC Co., Ltd., China) at ×40 lateral magnification (01.0-mm aperture).

### 3.19 RNA extraction from rat kidneys and qRT-PCR analysis

Rat kidneys (60 mg) stored at −80°C were lysed with 500 μL of lysis solution. The tissues were homogenized using an electric high-speed homogenizer. Total RNA was extracted from the lysate using an RNA isolation kit. The RNA purity was determined using the SmartSpec Plus nucleic acid and protein analyzer. The total RNA isolated from kidneys was reverse-transcribed using a PrimeScript RT Reagent Kit with a gDNA eraser (perfect real-time) and a qRT-PCR instrument. qRT-PCR analysis was performed under the following conditions: pre-denaturation at 95°C for 3 min, followed by 40 cycles of denaturation (at 94°C for 10 s), annealing (at 55°C for 30 s), and extension (at 72°C for 2 min). GAPDH was used as the internal reference gene. The relative gene expression levels were calculated using the 2^−ΔΔCT^ method. The primers were synthesized by Shanghai Shenggong Biological Engineering Co., Ltd. The intron-spanning primer sequences are shown in Table 3.

**TABLE 3:** Sequence of primers for rat kidney genes.

Gene name	Upper/lower primer	Sequence
GLUT9	Forward	5′-CTT​GCC​CTA​GCT​TCC​CTG​ATA​C-3′
	Reverse	5′-TAG​AGG​AAG​GAG​GAC​CCG​AAG-3′
OAT1	Forward	5′-ACC​TTG​TGT​GCT​CTC​ATC​GG-3′
	Reverse	5′-TAG​TTG​GGT​GCA​TAG​GCT​GC-3′
OAT3	Forward	5′-CCT​GAA​GAC​ACT​CCA​ACG​GG-3′
	Reverse	5′-ACA​CGA​CGA​AGG​ATG​GAC​AC-3′
GAPDH	Forward	5′-GGT​TGT​CTC​CTG​CGA​CTT​CA-3′
	Reverse	5′-TGG​TCC​AGG​GTT​TCT​TAC​TCC-3′

### 3.20 Immunofluorescence analysis of rat kidney sections

The rat kidney sections on glass slides were dewaxed in xylene, dehydrated by washing in absolute ethanol, and washed in water for 3–5 min. The sections were then probed with anti-GLUT9 (1:5,000) (Cat #67530-1-Ig; Proteintech Co., Ltd.), anti-OAT1 (1:1,000) (Cat #DF8582; Affinity Co., Ltd.), or anti-OAT3 (1:500) antibody (Cat #AF5749; Affinity Co., Ltd.) for 1 h, followed by rinsing thrice with PBS-T. Next, the sections were incubated with FITC-conjugated secondary antibody (1:300) (Cat #09JUL2020; Millipore Co., Ltd.) for 1 h. The sections were mounted with mounting medium (80312-3181; Jiangsu Sitai Experimental Equipment Co., Ltd.) containing the fluorescent dye DAPI for nuclear staining. The fluorescence signals of GLUT9, OAT1, or OAT3 were detected using a fluorescence microscope (XSP-C204; CIC Co., Ltd.) at ×40 lateral magnifications (01.0-mm aperture).

### 3.21 Statistical analysis

SPSS 25.0 software was used for statistical analysis. The data are expressed as mean ± standard errors. Means between multiple groups were compared using one-way analysis of variance, followed by least significant difference test (for data with uniform variance) or Dunnett’s T3 test (for data with non-uniform variance). Differences were considered significant at *p* < 0.05.

## 4 Results

### 4.1 DTX treatment improves the overall health of HUA quails

During the study period, quails in the control group were in good health and exhibited typical dietary habits (physiological activity, standard coat color, healthy spirit, and healthy feces). In contrast, quails in the HUA model group exhibited reduced activity, dull coat color, reduced appetite, and depressed spirit. In the DTX-treated groups of quails (DTX treated at doses of 1.8, 3.6, and 7.2 g/kg bodyweight), the overall health condition improved, and the quails exhibited good spirit, smooth coat color, and healthy feces and dietary habits.

### 4.2 DTX treatment decreases the levels of UA, XOD, Cre, and Bun in the serum of HUA quails

XOD catalyzes the production of UA (So and Thorens, 2010). The upregulation of XOD activity accelerates the production of UA, enhancing the levels of UA (Stamp et al., 2021). Cre and BUN are sensitive indicators of renal function (Nakada et al., 2023; Zeng et al., 2023). To investigate the effects of DTX on XOD activity, UA level, and renal function in the HUA quail model, the serum levels of UA, XOD, Cre, and BUN were examined on days 14, 21, 28, and 35.

On day 14, compared with those in the control group, the UA, XOD, BUN, and Cre levels were significantly higher in the HUA model group (*p* < 0.05 or *p* < 0.01) (Figures 1C–F). Conversely, the UA, XOD, BUN, and Cre levels in the high-dose DTX (7.2 g/kg bodyweight) group, the XOD levels in the medium-dose DTX (3.6 g/kg bodyweight), and the Cre levels in the low-dose DTX (1.8 g/kg bodyweight) group were significantly lower than those in the HUA model group (*p* < 0.05) (Figure 1D−E). The UA, BUN, and Cre levels in the medium-dose DTX (3.6 g/kg bodyweight) group and the UA, XOD, and BUN levels in the low-dose DTX (1.8 g/kg bodyweight) group were not significantly lower than those in the HUA model group (*p* > 0.05) (Figures 1C,D,F).

On day 21, compared with those in the control group, the UA, XOD, BUN, and Cre levels were significantly higher in the HUA model group (*p* < 0.05 or *p* < 0.01) (Figures 1G–J). Compared with those in the HUA model group, the UA, XOD, BUN, and Cre levels were significantly lower in the high-dose DTX (7.2 g/kg bodyweight) group; the UA, XOD, and Cre levels were significantly lower in the medium-dose DTX (3.6 g/kg bodyweight) group; and the BUN levels were significantly lower in the low-dose DTX (1.8 g/kg bodyweight) group (*p* < 0.05) (Figures 1G–J). The BUN levels in the medium-dose DTX (3.6 g/kg bodyweight) group and the UA, XOD, and Cre levels in the low-dose DTX (1.8 g/kg bodyweight) group were not significantly lower than those in the HUA model group (*p* > 0.05) (Figures 1G–J).

On day 28, compared with those in the control group, the UA, XOD, BUN, and Cre levels were significantly higher in the HUA model group (*p* < 0.05 or *p* < 0.01) (Figure 1K–N). Compared with those in the HUA model group, the UA, XOD, BUN, and Cre levels were significantly lower in the medium-dose DTX (3.6 g/kg bodyweight) and high-dose DTX (7.2 g/kg bodyweight) groups, and the UA and Cre levels were significantly lower in the low-dose DTX (1.8 g/kg bodyweight) group (*p* < 0.05 or *p* < 0.01) (Figure 1K–M). The XOD and BUN levels in the low-dose DTX (1.8 g/kg bodyweight) group were not significantly lower than those in the HUA model group (*p* > 0.05) (Figures 1L,N).

On day 35, the UA, XOD, BUN, and Cre levels in the HUA model group were significantly higher than those in the control group (*p* < 0.05 or *p* < 0.01) (Figure 1O–R). Compared with those in the HUA model group, the UA, XOD, BUN, and Cre levels were significantly lower in the low-dose DTX (1.8 g/kg bodyweight), medium-dose DTX (3.6 g/kg bodyweight), and high-dose DTX (7.2 g/kg bodyweight) groups (*p* < 0.05 or *p* < 0.01) (Figure 1O–R).

### 4.3 DTX treatment increases the fecal excretion of UA in the HUA quail model

**FIGURE 2:**
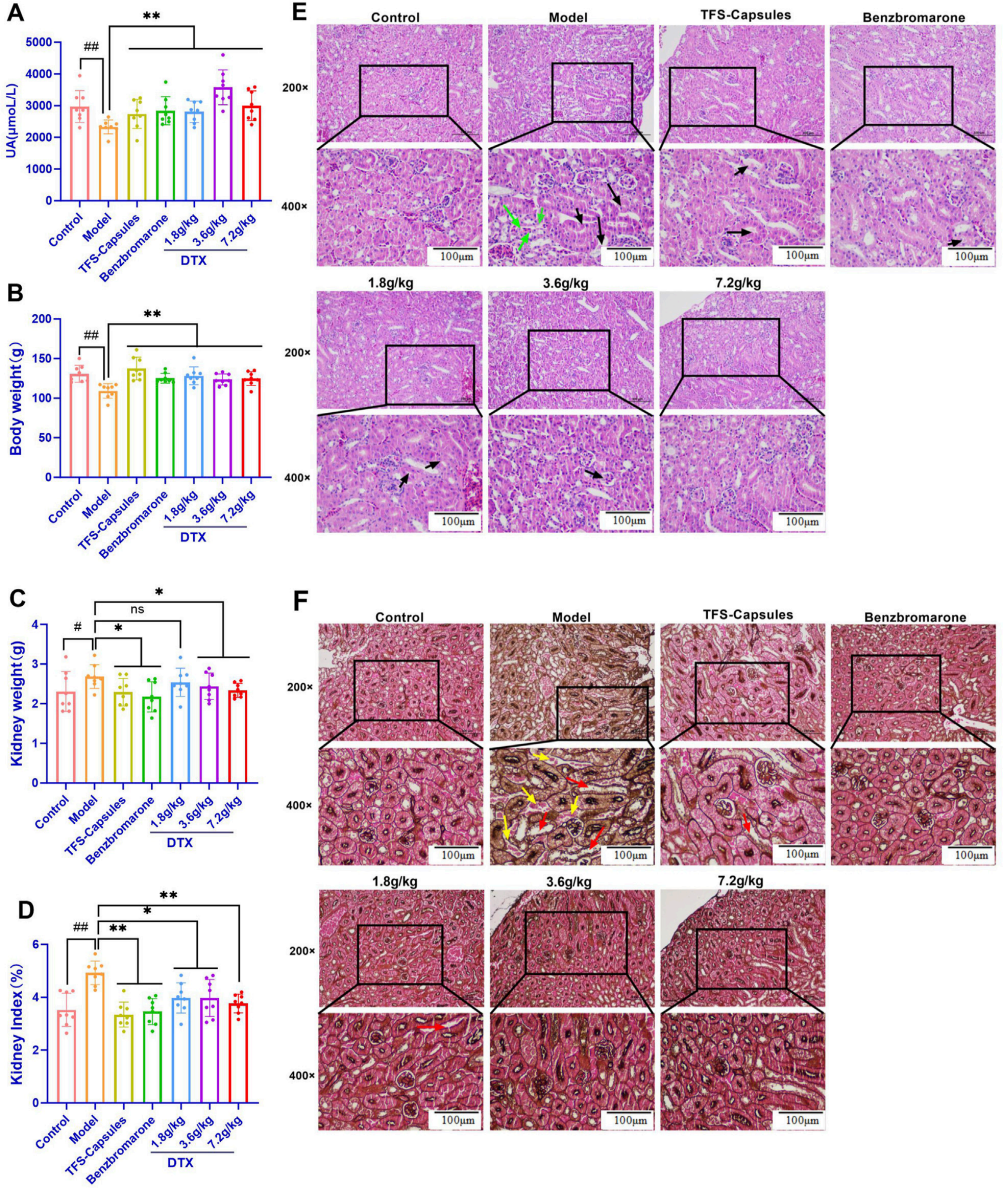
DTX increases bodyweight and decreases kidney weight and the renal index in the HUA quail model (n = 8) and improves the pathomorphology of quail kidneys (n = 3). In the control, HUA, and drug-treated HUA quails, the following items were measured on day 35: **(A)** fecal UA content, **(B)** body weight (average), **(C)** weight of the kidneys, and **(D)** kidney index. **(E)** The representative photomicrographs show H&E staining of kidney sections visualized at ×200 and ×400 magnifications; green arrow indicates focal tubular shrinkage, and black arrow indicates detachment of tubular epithelial cells; **(F)** hexamine Ag staining of kidney sections visualized at ×200 and ×400 magnifications. Red arrows indicate the protein tubular pattern, while yellow arrows indicate brush border damage/detachment of renal tubular epithelial cells. ^#^ *p* < 0.05 and ^##^ *p* < 0.01 compared with the control group. ^*^ *p* < 0.05 and ^**^ *p* < 0.01 compared with the model group*;* ns: *p* > 0.05.

Compared with the control group, the fecal excretion of UA was significantly reduced in the HUA model group (*p* < 0.01) (Figure 2A). DTX treatment (1.8, 3.6, or 7.2 g/kg bodyweight) significantly increased the fecal excretion of UA in the HUA quail model (*p* < 0.01) (Figure 2A).

### 4.4 DTX treatment improves the bodyweight and kidney index in the HUA quail model

The health status of quails was evaluated based on their bodyweight and kidney index values. Compared with those in the control group, the bodyweight was significantly lower and the kidney weight and kidney index values were significantly higher in the HUA model group (*p* < 0.05 or *p* < 0.01) (Figures 2B–D). Conversely, compared with those in the HUA model group, the bodyweight of quails was significantly higher and the kidney weight and kidney index values were significantly lower in the medium-dose DTX (3.6 g/kg bodyweight) and high-dose DTX (7.2 g/kg bodyweight) groups (*p* < 0.05 or *p* < 0.01) (Figures 2B–D). The bodyweight was significantly higher and the renal index values were significantly lower in the low-dose DTX (1.8 g/kg bodyweight) group (*p* < 0.05 or *p* < 0.01) (Figure 2B).

### 4.5 DTX ameliorates glomerular structural changes and renal inflammatory response in the HUA quail model

As yeast powder is rich in proteins and nucleotides, it is commonly used to induce UA production. A high intake of a solution/suspension of sterile dry yeast powder can increase UA levels and promote UA crystal deposition in the kidney, inflammatory cell infiltration, tissue hyperplasia, HUA formation, and renal dysfunction (Zhou et al., 2010). The renal inflammatory response and glomerular structural changes were examined using HE staining. In the control group, intraglomerular and intratubular eosinophilic epithelial cells exhibited focal and lamellar distribution in the kidney, the glomeruli and tubules exhibited physiological morphology, the tubules did not undergo degeneration, and brush border detachment was not observed (Figure 2E). Compared with those in the control group, the intraglomerular and intratubular eosinophilic epithelial cells were focally distributed, focal tubules were narrow, and the epithelial cells of the tubules were detached in the HUA model group (Figure 2E). Conversely, compared with those in the HUA model group, some renal tubular epithelial cells were separated at the brush border, epithelial cells were not detached, tubular formation and inflammatory cell infiltration were not observed in the surrounding area, the glomerular morphology and structure were clear, and no notable pathological changes were observed in the DTX-treated groups of HUA quails (Figure 2E).

### 4.6 DTX restores glomerular structural changes, renal inflammatory response, and renal fibrosis in the HUA quail model

The renal tubules of quail kidneys in the control group did not exhibit degeneration, explicit tubular configuration, and brush border detachment (Figure 2F). Compared with those in the control group, the detachment of several epithelial cells in the renal tubules from the brush border, protein tubular configuration, and tubular dilatation were observed in the HUA model group (Figure 2F). Conversely, compared with those in the HUA model group, only a few epithelial cells exhibited brush border detachment in the DTX treatment groups. Additionally, the interstitium of the kidney did not have inflammatory cell infiltration, fibrous tissue, and proliferative tubular configuration, and the structure and glomerular morphology were more physiological in the DTX treatment group (Figure 2F).

### 4.7 DTX improves the overall health of the HUA rat model

The results of this study demonstrated that compared with those in the control group, the rats in the HUA model developed rough fur and bulging abdomens on both sides, which were accompanied by shrugging, cuddling, and curling behaviors; loosening; reduced appetite and activity; and depression. Conversely, compared with those in the HUA model group, the rats in the DTX-treated groups exhibited physiological hair texture, increased activity, and no clumping and curling behaviors.

### 4.8 DTX increases the 24-h urine volume, 24-h UA excretion, and UA excretion fraction in the HUA rat model

**FIGURE 3:**
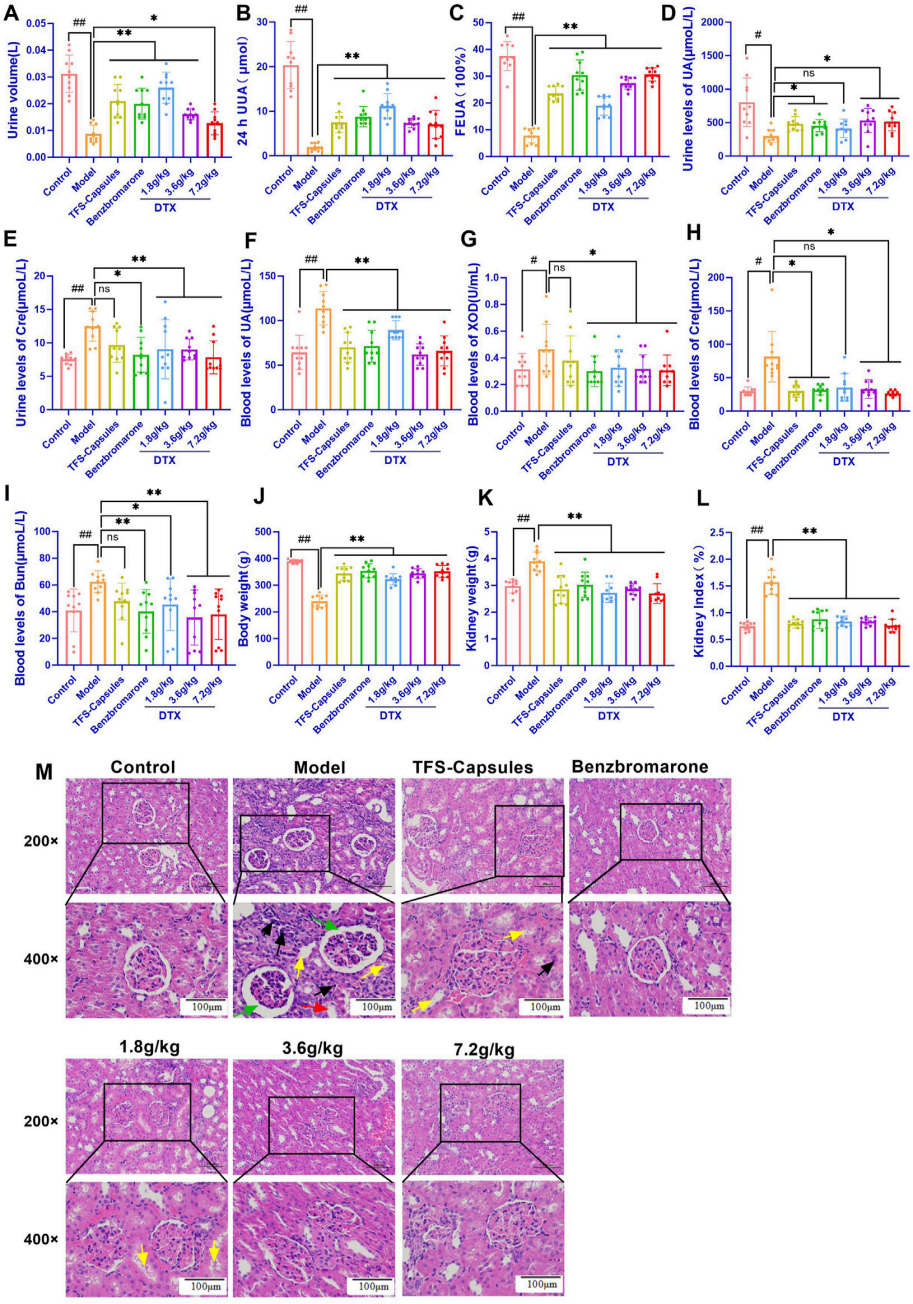
In the control, HUA, and drug-treated HUA rats, the following parameters were measured on day 35: **(A)** 24-h urine volume, **(B)** 24-h UA excretion, **(C)** UA excretion fraction in the rat HUA model, **(D)** level of urinary UA, **(E)** level of urinary creatinine (Cre), **(F)** serum level of UA, **(G)** serum xanthine oxidase (XOD) activity, **(H)** serum level of Cre, **(I)** serum level of BUN, **(J)** body weight, **(K)** kidney weight, **(L)** and kidney index. **(M)** Representative photomicrographs show H&E staining (200× and 400×) (n = 3). Green arrows indicate dilated glomerular Bowman’s capsule lumen; red arrows indicate epithelial cell detachment; yellow arrows indicate the unstructured exudate and cellular tubular pattern visible in the tubular lumen; and black arrows indicate small foci of lymphocytic infiltration into the interstitium. #*p* < 0.05 and ##*p* < 0.01 compared with the control group. *p < 0.05 and **p < 0.01 compared with the model group.

The 24-h UA excretion volume and UA excretion fraction can accurately measure the level of UA excretion by the kidney. Compared with those in the control group, the 24-h urine volume, 24-h UA excretion, and UA excretion fraction were significantly reduced in the HUA model group (*p* < 0.01) (Figures 3A–C). Conversely, compared with those in the HUA model group, the 24-h urine volume, 24-h UA excretion, and UA excretion fraction were significantly higher in the low-dose DTX (1.8 g/kg bodyweight), medium-dose DTX (3.6 g/kg/bodyweight), and high-dose DTX (and 7.2 g/kg bodyweight) groups (*p* < 0.05 or *p* < 0.01) (Figures 3A–C).

### 4.9 DTX reduced the serum UA, XOD, Cre, and BUN levels and increased urinary UA excretion in the HUA rat model

Serum XOD activity is an important index for HUA diagnosis (Tang et al., 2017). SU can be used to evaluate the degree of pathogenesis of HUA. Cre and BUN are sensitive indicators for assessing renal function (Nakada et al., 2023; Zeng et al., 2023). In this study, compared with those in the control group, the urinary UA levels were significantly decreased and the Cre levels were significantly increased in the HUA model group (*p* < 0.05 or *p* < 0.01) (Figures 3D–H). Conversely, compared with those in the HUA model group, the urinary UA levels were significantly higher and the Cre levels were significantly lower in the medium-dose DTX (3.6 g/kg bodyweight) and high-dose DTX (7.2 g/kg bodyweight) groups, and the Cre levels were significantly decreased in the low-dose DTX (1.8 g/kg bodyweight) group (*p* < 0.05 or *p* < 0.01) (Figures 3D–H). The urinary UA levels in the low-dose DTX (1.8 g/kg bodyweight) group were not significantly different from those in the HUA model group (*p* > 0.05) (Figure 3D).

### 4.10 DTX treatment improves kidney morphology in the HUA rat model

The kidneys of rats in the control group were intact with reddish and smooth surfaces and exhibited physiological morphology. Compared with those of rats in the control group, the kidneys of rats in the HUA model group were enlarged and exhibited uneven texture and white nodule-like changes, suggesting severe kidney lesions. Conversely, compared with those of rats in the HUA model group, the kidneys of rats in the three DTX-treated groups exhibited a flatter surface, softer texture, bright red color, and lower white nodule-like changes.

### 4.11 DTX treatment decreases kidney weight and the ratio of kidney weight to body weight in the HUA rat model

The ratio of kidney weight to bodyweight is one of the important indexes that indicates renal function (Kim et al., 2001). Compared with those in the control group, the bodyweight was significantly lower and the kidney weight and renal index values (ratio of the kidney weight to bodyweight) were significantly higher in the HUA model group (*p* < 0.01) (Figure 3I–L). Conversely, compared with those in the HUA model group, the bodyweight was significantly higher and the kidney weight and renal index values were significantly lower in the low-dose DTX (1.8 g/kg bodyweight), medium-dose DTX (3.6 g/kg bodyweight), and high-dose DTX (7.2 g/kg bodyweight) groups (*p* < 0.01) (Figure 3I–L).

### 4.12 DTX restores glomerular structural changes and renal inflammatory response in the HUA rat model

Adenine increases UA biosynthesis, while ethambutol hydrochloride (an antituberculosis agent) decreases the renal clearance of UA by the kidney, leading to deposition of UA crystals in the kidneys, infiltration of inflammatory cells, tissue hyperplasia, HUA development, and renal dysfunction (Sui et al., 2023). Renal inflammatory changes and glomerular structural changes were examined using HE staining. In the control group, rat kidneys exhibited physiological cellular morphology and glomerular size and morphology with no hyperplasia and atrophy. Additionally, the dilation of renal tubules and Bowman’s capsule lumen and the infiltration of inflammatory cells were not observed (Figure 3M). Compared with those in the control group, the lumen of glomerular Bowman’s capsule and renal tubules was dilated, the tubular epithelium exhibited vacuolar degeneration, some epithelial cells were detached, and the tubular lumen exhibited unstructured exudate and cellular tubular patterns in the HUA model group. Other important notable pathological features of the HUA quail kidneys include some small foci of infiltration of lymphocytes into the interstitium (Figure 3M). Conversely, compared with those in the HUA model group, the lumen of glomerular Bowman’s capsule and renal tubules was not dilated, some protein tubular formation was observed in individual tubules, inflammatory cells around the periphery were not observed, the glomerular structure was clear, and no notable pathological changes were observed in the DTX treatment groups (Figure 3M).

### 4.13 DTX restores the relative gene expression of GLUT9, OAT1, and OAT3 in HUA quail kidneys

**FIGURE 4:**
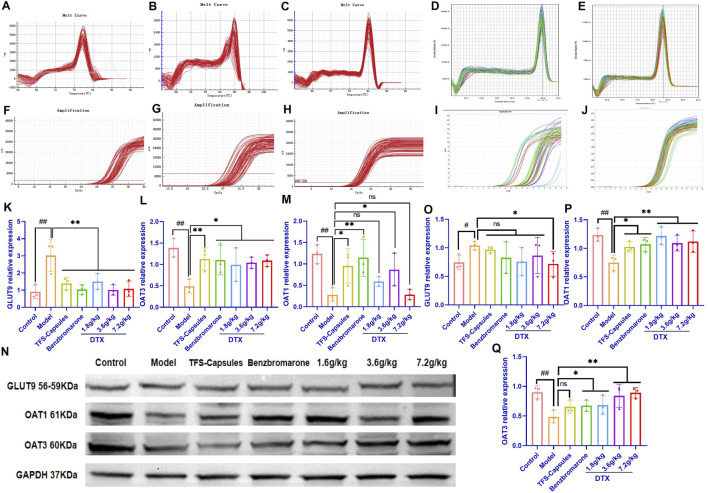
DTX treatment decreased the expression of GLUT9, OAT1, and OAT3 in the HUA quail kidneys (n = 3). The melting curves of RT-PCR products of renal **(A)** GLUT9, **(B)** OAT3, **(C)** GAPDH, **(D)** OAT1, and **(E)** β-actin in the quail kidneys. The amplification curve of the RT-PCR products of **(F)** GLUT9, **(G)** OAT3, **(H)** GAPDH, **(I)** OAT1, and **(J)** β-actin in the quail kidneys. The bar diagram showing the relative expression levels of **(K)** GLUT9 mRNA, **(L)** OAT3 mRNA, and **(M)** OAT1 mRNA. **(N)** Results of Western blotting showing the relative expression levels of GLUT9, OAT1, OAT3, and GAPDH in the kidneys of control, HUA model, and drug-treated quails. An equal amount (75 µg/lane) of total protein was loaded in each lane of 10% SDS-polyacrylamide gel, fractionated, and then analyzed. The bar diagram shows the relative expression levels of the **(O)** GLUT9 protein, **(P)** OAT1 protein, and **(Q)** OAT3 protein in the quail kidneys. Relative expression was estimated after normalizing with GAPDH/β-actin. #*p* < 0.05 and ##*p* < 0.01 compared with the control group. *p < 0.05 and **p < 0.01 compared with the model group; ns: *p* > 0.05.

To examine the relative mRNA expression levels of GLUT9, OAT1, OAT3, GAPDH, and β-actin in the control, HUA model, and DTX-treated groups, the qRT-PCR products were subjected to melting curve analysis to check the specificity of amplification (Figures 4A–E). Next, the amplification curves of the GLUT9, OAT1, OAT3, GAPDH, and β-actin qRT-PCR products (Figures 4F–J) from the kidneys of the control, HUA model, and DTX-treated quails were examined to calculate the respective Ct values. The results of qRT-PCR analysis (after normalization of the expression of target genes with that of GAPDH/β-actin) demonstrated that compared with those in the control group, the GLUT9 mRNA levels were significantly increased (Figure 4K) and the OAT1 (Figure 4L) and OAT3 (Figure 4M) mRNA levels were significantly decreased (*p* < 0.05 or *p* < 0.01) in the HUA model group. However, treatment with DTX (1.8, 3.6, or 7.2 g/kg bodyweight) significantly decreased the GLUT9 mRNA expression level (*p* < 0.05) (Figure 4K) and significantly increased the OAT1 (Figure 4M) and OAT3 (Figure 4L) mRNA expression levels (*p* < 0.05).

DTX treatment downregulated the renal expression of the GLUT9 protein and increased the renal expression of OAT1 and OAT3 proteins in the HUA quail model. To analyze the correlation between mRNA and the protein expression levels, the relative protein expression levels of GLUT9, OAT1, OAT3, and GAPDH in the kidneys of the control, model HUA, and DTX-treated HUA quails were analyzed using Western blotting (Figure 4N–Q). The results of Western blotting demonstrated that compared with those in the control group, the GLUT9 protein levels were significantly increased and the OAT1 and OAT3 protein levels were significantly decreased in the HUA model group (*p* < 0.05 or *p* < 0.01) (Figure 4N–Q). Conversely, compared with those in the HUA model group, the OAT1 and OAT3 protein levels were significantly increased in the low-dose DTX (1.8 g/kg bodyweight), medium-dose DTX (3.6 g/kg bodyweight), and high-dose DTX (7.2 g/kg bodyweight) groups, and the GLUT9 protein levels were significantly decreased in the high-dose DTX (7.2 g/kg bodyweight) group (*p* < 0.05 or *p* < 0.01) (Figure 4N–Q). The GLUT9 protein levels in the low-dose DTX (1.8 g/kg bodyweight) and medium-dose DTX (3.6 g/kg bodyweight) groups were not significantly lower than those in the HUA model group (*p* > 0.05) (Figure 4N–O).

### 4.14 DTX treatment downregulates the *in situ* expression of GLUT9 protein and upregulates the *in situ* expression of OAT1 and OAT3 proteins in HUA quail kidneys

**FIGURE 5:**
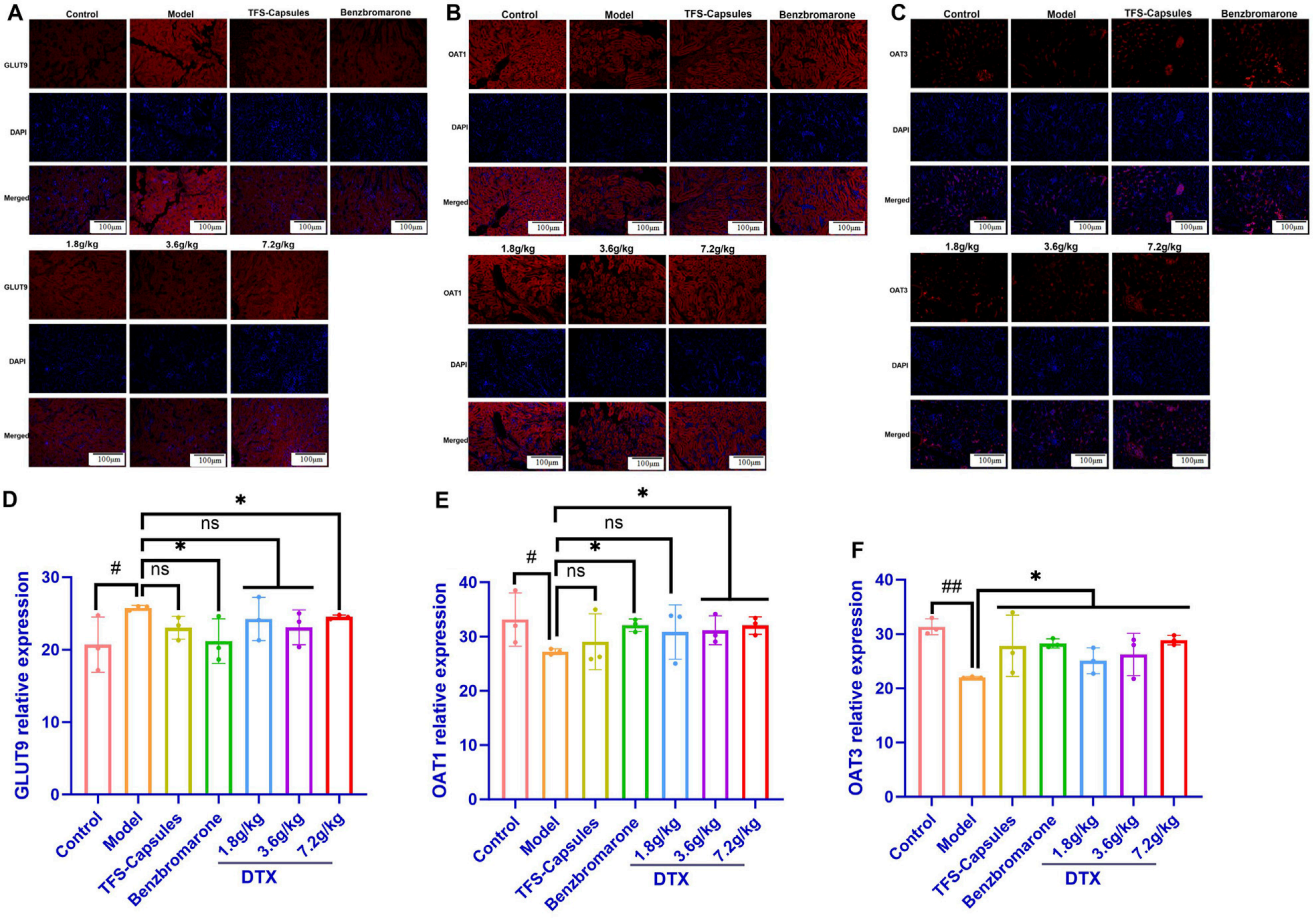
DTX decreased GLUT9 protein expression and increased OAT1 and OAT3 protein expression in HUA quail kidneys (n = 3). The representative photomicrographs showing immunodetection of the expression of the **(A)** GLUT9 protein, **(B)** OAT1 protein, and **(C)** OAT3 protein in the kidney sections of control, HUA quails, and DTX-treated HUA quails. Measurement of the mean fluorescent intensity across the selected region of each subfigure shows the relative expression level of GLUT9 **(D)**, OAT1 **(E)**, and OAT3 **(F)** in the quail kidneys. Red: GLUT9 protein; blue: 4,6-diamino-2-phenylindole (DAPI). Compared with the control group, #*p* < 0.05 and ##*p* < 0.01. Compared with the model group, *p < 0.05 and **p < 0.01; ns, not statistically significant: *p* > 0.05.

To further verify the relative expression levels of GLUT9, OAT1, and OAT3 *in situ*, the quail kidneys were subjected to immunofluorescence analyses. Compared with those in the control group, the OAT1 and OAT3 protein levels were lower and the GLUT9 protein levels were higher in the HUA model group (*p* < 0.05 or *p* < 0.01) (Figures 5A–F). Conversely, compared with those in the HUA model group, the OAT1 and OAT3 protein levels were significantly increased and the GLUT9 protein levels were significantly decreased in the high-dose DTX (7.2 g/kg bodyweight) group and the OAT1 and OAT3 protein levels were significantly increased in the medium-dose DTX (3.6 g/kg bodyweight) group. The OAT3 protein levels were significantly increased in the low-dose DTX (1.8 g/kg bodyweight) group (*p* < 0.05 or *p* < 0.01) (Figures 5A–F). Compared with those in the HUA model group, the GLUT9 protein levels and the OAT1 levels were not significantly increased in the medium-dose DTX (3.6 g/kg bodyweight) group. In the low-dose DTX (1.8 g/kg bodyweight) group, the GLUT9 protein levels were not significantly decreased (*p* > 0.05) (Figure 5E).

### 4.15 DTX restores the relative gene expression of GLUT9, OAT1, and OAT3 in HUA rat kidneys

**FIGURE 6:**
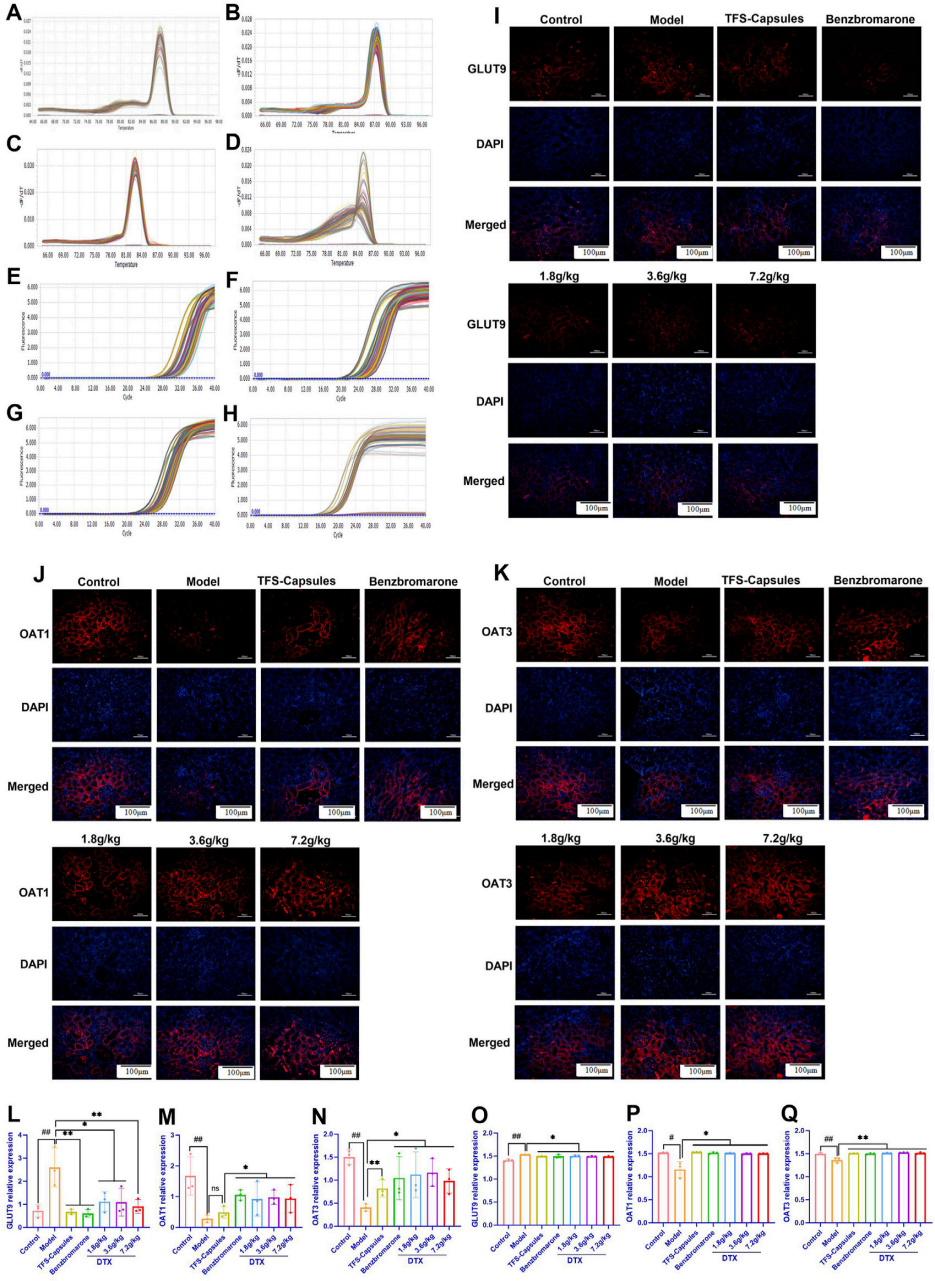
DTX decreased the expression of GLUT9 and increased the expression of OAT1 and OAT3 in rat kidneys (n = 3). The melting curve of the RT-PCR product of renal **(A)** GLUT9, **(B)** OAT1, **(C)** OAT3, and **(D)** GAPDH of rats. The amplification curve of the RT-PCR product of renal **(E)** GLUT9, **(F)** OAT1, **(G)** OAT3, and **(H)** GAPDH of rats. The representative photomicrographs showing the immunodetection of **(I)** GLUT9, **(J)** OAT1, and **(K)** OAT3 protein expression in rat kidney sections. The bar diagrams show the relative expression levels of renal **(L)** GLUT9 mRNA, **(M)** OAT1 mRNA, **(N)** OAT3 mRNA, **(O)** GLUT9 protein, **(P)** OAT1 protein, and **(Q)** OAT3 protein in control, HUA, and DTX-treated HUA rats. The mean fluorescent intensity was measured across the selected region of each subfigure of immunofluorescence. Compared with the control group, #*p* < 0.05 and ##*p* < 0.01. Compared with the model group, *p < 0.05 and **p < 0.01.

To examine the relative mRNA expression levels of GLUT9, OAT1, OAT3, and GAPDH in the control, HUA model, and DTX-treated rats, the qRT-PCR products were subjected to melting curve analysis to check the specificity of amplification (Figures 6A–D). Next, the amplification curves of the GLUT9, OAT1, OAT3, and GAPDH qRT-PCR products from the kidneys (Figures 6E–H) of the control, model HUA, and DTX-treated HUA rats were analyzed to calculate the respective Ct values. The Ct values of GLUT9, OAT1, OAT3, and GAPDH mRNAs in the rat kidneys were between 10 and 35. The results of the qRT-PCR analysis (after normalization of the expression of target genes with that of GAPDH) demonstrated that compared with those in the control group, the GLUT9 mRNA levels were significantly increased (Figure 6L) and the OAT1 (Figure 6M) and OAT3 (Figure 6N) mRNA levels were significantly decreased (*p* < 0.05 or *p* < 0.01) in the HUA model group. However, treatment with DTX (1.8, 3.6, or 7.2 g/kg bodyweight) significantly decreased the GLUT9 mRNA expression level (*p* < 0.05) (Figure 6L) and significantly increased the OAT1 (Figure 6M) and OAT3 (Figure 6N) mRNA expression levels (*p* < 0.05 or *p* < 0.01).

DTX treatment downregulates the *in situ* expression of the GLUT9 protein and upregulates the *in situ* expression of OAT1 and OAT3 proteins in the HUA rat kidneys. To further verify the relative expression levels of GLUT9, OAT1, and OAT3 *in situ*, the rat kidneys were subjected to immunofluorescence analyses. Compared with those in the control group, the renal OAT1 and OAT3 protein levels were significantly decreased and the renal GLUT9 protein levels were significantly increased in the HUA model group (*p* < 0.05 or *p* < 0.01) (Figures 6I–K and 6O–Q). Compared with the HUA model group, the OAT1 and OAT3 protein levels were significantly increased and the GLUT9 protein levels were significantly decreased in the high-dose DTX (7.2 g/kg bodyweight), medium-dose DTX (3.6 g/kg bodyweight), and low-dose DTX (1.8 g/kg bodyweight) groups (*p* < 0.05 or *p* < 0.01) (Figures 6I–K, O–Q).

## 5 Discussion

HUA is a metabolic disease characterized by excessive SU concentrations due to increased biosynthesis of urate and/or reduced excretion of urate. Gout, which is the most prevalent type of debilitating inflammatory arthritis worldwide, is caused by the deposition of monosodium urate (MSU) crystals in articular (especially the metatarsal and phalangeal joints) and non-articular structures (e.g. tendons, ligaments, burse, and even muscles). SU upregulation (HUA) is the most important risk factor for the development of acute and chronic inflammatory gout, urinary tract stones, and renal lesions (Estiverne and Mount, 2020). Innate immunity plays a pivotal role in gout development and progression. Various immune cells and inflammatory factors recognize MSU, initiating or exacerbating the inflammatory response by activating intracellular signaling pathways (Martinon et al., 2006; Shi Y et al., 2010). The treatment of millions of patients with HUA is a major challenge worldwide. Dai medicine has been developed from the accumulated experience of the Dai people in China in their fight against diseases after gaining knowledge from ancient Indian medicine and Chinese medicine with distinctive national and local characteristics (Tan et al., 2013). Additionally, Dai medicine in Yunnan, China, is a rich resource and has a wide range of clinical applications with good efficacy and limited side effects. The Dai medicine metabolite DTX is clinically effective in treating HUA (Liu et al., 2024).

UA is the end product of purine metabolism in the human body and is primarily excreted via the kidneys (So and Thorens, 2010). The production and excretion of UA in healthy individuals are in a dynamic balance. The dysregulation of this balance leads to increased production or decreased excretion of UA, resulting in upregulation of blood UA levels (Mandal and Mount, 2015; Estiverne and Mount, 2020). HUA has two main pathogenic mechanisms. The first mechanism is the excessive production of UA, which is mainly caused due to metabolic processes, although food intake plays a minor role (Yang et al., 2022). For example, the intake of purine-rich foods, such as broth, seafood, and beer, increases UA load in the body and exacerbates HUA as purine is the precursor of UA (Ding et al., 2021). Anabolic dysregulation accounts for disease onset in approximately 20% of patients (Pang et al., 2021). The upregulation of the expression or activity of ribose-phosphate pyrophosphokinase (RPS), XOD, and other intermediary metabolic enzymes involved in the synthesis of UA will indirectly or directly increase UA production (Lioté, 2019). Additionally, fructose intake was reported to increase SU levels. Adenosine phosphate may also enter the purine nucleotide degradation pathway, increasing SU levels (Mandal and Mount, 2015; Wang and Wang, 2020). The alternative pathogenic mechanism of HUA is decreased UA excretion. Approximately two-thirds of human UA is excreted via kidneys daily. This process involves glomerular filtration and tubular reabsorption. Insufficient renal urate excretory capacity increases the blood levels of UA, which is regulated by various urate transporters (Eraly et al., 2008; Park et al., 2019). For example, GLUT9 is the sole urate transporter that transports reabsorbed urate from the tubular epithelium to the blood. However, URAT1 promotes reabsorption of urate from the glomerular filtrate to the tubular epithelium in exchange for nicotinate or pyrazinoate (Mandal and Mount, 2015; Mandal et al., 2017). The organic anion transporters OAT1 and OAT3 (located at the basolateral membrane) are urate secretion transporters that transport urate from the blood to the tubular epithelium. Urate is finally secreted by apical urate transporters, such as ABCG2, ABCC4, NPT1, and NPT4 (Mandal and Mount, 2015; Mandal et al., 2017). Thus, OAT1 and OAT3 are essential urate transporters for urate secretion. The modulation of the expression of GLUT9, OAT1, and OAT3 can affect blood UA concentration.

Animal experiments are important for disease-related pharmacodynamic evaluation and mechanistic studies. The establishment of stable animal models is a prerequisite for experimental success. In this study, two animal models of poultry (quail) and rodents (rat) were selected to verify the pharmacodynamic effects of DTX. Quails belong to the family of birds that lack uricase like humans. Although rodents and humans have similar physiological and biochemical characteristics, rodents can convert UA into a soluble metabolite (allantoin) due to the activities of the enzyme urate oxidase/uricase (Mandal and Mount, 2015). The deposition of UA results in the formation of gouty stones. The serum UA level in humans is approximately 10 times higher than that in rodents (Bassi and Fattoruso et al., 2020). Although rodents are not an ideal model, their small size, ease of feeding and reproduction, low price, and high sensitivity to drugs are advantageous for the establishment of disease models. The UA level in female rodents is affected by external interference and fluctuations, which may lead to fluctuations in hormone secretion. The physiological profiles of male rats are more stable than those of female rats (Zhu et al., 2017). In this study, quails and rats were selected to explore the pharmacodynamic effects and anti-hyperuricemic mechanisms of DTX.

In this study, a solution/suspension of sterile yeast extract powder (containing many amino acids and nucleotides) was administered by gavage feeding to induce HUA in quails (overnutrition-induced HUA). Yeast extract can increase the activity of XOD and upregulate the UA levels to modulate homeostasis during physiological purine metabolism (Zhu et al., 2017). Some studies have demonstrated that yeast dry powder-induced HUA in quails may be related to the inhibition of the glycolytic pathway, activation of the pentose phosphate pathway, and XOD (Zhang and Pope, 2017; Ovseychik et al., 2024). In rats, HUA was induced by gavage feeding a solution of adenine and ethambutol hydrochloride. Adenine is a precursor of UA synthesis, while ethambutol hydrochloride can inhibit urate excretion. Ethambutol hydrochloride can also cause nephrotoxicity to a certain degree (Zhu Y et al., 2017).

XOD oxidizes hypoxanthine to xanthine and, subsequently, to UA. The upregulation of XOD activity accelerates the production of UA (Chen et al., 2022). DTX significantly decreased the serum level of XOD, suggesting that DTX can reduce the conversion of xanthine to UA. The serum and urinary levels of UA can be used to evaluate the degree of HUA development. This study demonstrated that DTX decreased UA levels in the two HUA animal models and increased urinary excretion of UA in the rat HUA model and fecal excretion of UA in the quail HUA model. These findings suggest that the anti-hyperuricemic mechanism of DTX involves the upregulation of urinary UA excretion and the downregulation of the SU level. Additionally, this study demonstrated the renal protective effects of DTX. Cre and BUN, which are the metabolites of proteins that are mainly excreted by the kidneys via glomerular filtration (Ruan et al., 2023), are sensitive indicators to renal function. The upregulation of BUN and Cre levels in the blood is indicative of renal dysfunction (Ruan et al., 2023). In this study, the Cre and BUN levels were significantly upregulated in the HUA model group, indicating renal dysfunction in HUA model animals. DTX suppressed the levels of Cre and BUN in the HUA model, suggesting that DTX alleviates renal dysfunction.

Adenine and ethambutol hydrochloride can damage the renal tissues at specific doses, inhibiting the ability of the kidneys to physiologically metabolize and excrete UA. This leads to the upregulation of serum UA levels, deposition of UA crystals in the kidneys, infiltration of inflammatory cells, and tissue hyperplasia, resulting in HUA development and renal dysfunction (Zhu et al., 2017). Gavage feeding of adenine + ethambutol hydrochloride can impair the typical morphology, color, and surface of the kidneys, which is consistent with the clinical physiological and structural changes in the kidneys of patients with HUA (Kang et al., 2020). DTX ameliorates the changes in the kidney morphology and color and alleviates kidney damage. The renal index is one of the important indexes of renal function. In this study, the renal index was significantly increased in the HUA model animals (quails and rats) but was markedly decreased in the DTX treatment groups, suggesting that DTX exerts protective effects on the kidney. Analysis of 24-h UA excretion and UA excretion fraction can accurately assess the renal UA excretion capability/status. The UA excretion fraction refers to the percentage of UA filtered through the glomerulus that is finally excreted through urine (Ueno, 2017). Some studies (Baum et al., 1975) have demonstrated that the UA excretion fraction is related to the structural and functional aberrations of the renal tubule, reflecting the ability of the renal tubule to process UA. The accurate assessment of 24-h UA excretion and UA excretion fraction is critical for the assessment of renal health. DTX significantly increased the 24-h UA excretion and the UA excretion index, which suggested that the anti-hyperuricemic effect of DTX involves the renal protective properties of DTX.

Pathological analysis is the gold standard for analyzing disease pathogenesis. The experimental results of this study demonstrated successful establishment of the HUA model and the therapeutic effects of the drug on renal tissue damage at the cellular level. Hexamine Ag and HE staining of quail kidneys revealed that the renal tubules in the HUA model group exhibited shedding of renal tubular epithelial cells from the brush border, visible protein tubular pattern, tubular dilatation, and inflammatory cell infiltration. In contrast, DTX suppressed the shedding of renal tubular epithelial cells from the brush border, inhibited the formation of tubular patterns and inflammatory cell infiltration, and improved glomerular morphology and structure. HE staining of the kidney sections of the HUA rat model revealed the dilatation of the glomerular Bowman’s capsule lumen and renal tubules, vacuolar degeneration in the tubular epithelium, detachment of some epithelial cells, presence of a structureless exudate and cellular tubular pattern in the tubular lumen, and small foci of lymphocyte infiltration in the interstitium. In contrast, DTX treatment significantly decreased the inflammatory response of the kidney in the rats, improved the altered cellular structure, and reduced cellular infiltration into the interstitium. The UATG consisting of URAT1, GLUT9, OAT4, OAT10, OAT1, OAT3, ABCG2, ABCC4, NPT1, and NPT4 plays a crucial role in maintaining urate homeostasis because most blood urate (approximately 99%) is freely filtered through the glomerulus. However, approximately 90% of the filtered urate is reabsorbed via the urate transporters URAT1, OAT10, OAT4, and GLUT9, while only 10% of the filtered urate is excreted through the urine. SU can be secreted via OAT1 and OAT3 from the blood into the renal tubular epithelium and subsequently secreted via ABCG2, ABCC4, NPT1, and NPT4 from the renal tubular epithelium into the lumen of renal tubules for excretion (Mandal and Mount, 2015). The results of qRT-PCR, immunoblotting, and immunofluorescence analyses revealed that the mRNA and protein expression levels of GLUT9 in the HUA model animals were significantly downregulated after DTX treatment. In contrast, the expression levels (both mRNA and protein) of urate secretion transporters OAT1 and OAT3 were markedly upregulated after DTX treatment in the HUA model animals. Thus, we postulate that DTX facilitates urinary excretion of UA by downregulating the tubular reabsorption of UA via GLUT9 and upregulating the secretion of UA via OAT1 and OAT3 in the kidneys.

## 6 Conclusion

The results of this study suggest that DTX can exert anti-HUA effects by promoting UA excretion through the downregulation of GLUT9 expression and the upregulation of OAT1 and OAT3 expression. Additionally, DTX protects renal function by attenuating renal injury. Thus, DTX has potential clinical significance in treating HUA.

## Data Availability

The original contributions presented in the study are included in the article/Supplementary Material; further inquiries can be directed to the corresponding authors.

## Abbreviations

Ag, silver; BUN, blood urea nitrogen; Cre, creatinine; CVD, cardiovascular disease; DAPI, 4′,6-diamidino-2-phenylindole; DTX, daitongxiao; GLUT9, glucose transporter 9; H&E, hematoxylin and eosin; HUA, hyperuricemia; RT-PCR, real-time polymerase chain reaction; TFS capsules, Tongfengshu capsules; UA, uric acid; UATG, uric acid transporter group; UUA, urinary UA; XOD, xanthine oxidase.
