# Supplementary material for: Ameliorative action of “daitongxiao” against hyperuricemia includes the “uric acid transporter group”
Source: Front Pharmacol. 2024 Jun 25;15:1300131. doi: 10.3389/fphar.2024.1300131 (PMC11232504; doi:10.3389/fphar.2024.1300131)
Supplement: Supplementary file 1 [file Table1.docx]

**Supplementary Table S1.**

Profile of main chemical composition

| Botanical name | metabolite | References |
| --- | --- | --- |
| Elsholtzia rugulosa Hemsl. | Apigenin 4’-O-α-D-glucopyranoside | (She et al., 2009) |
|  | Glucopyranoside | (She et al., 2009) |
|  | 5,7,3',4'-tetrahydroxy-5'-C-prenylflavone-7-O-β-D-glucopyranoside | (She et al., 2009) |
|  | maltol 6’-O-β-D-apiofuranosyl-β-D-glucop-yranoside | (Li et al., 2007) |
|  | Amygdalin | (Li et al., 2007) |
|  | Methyl 2-O-(3,4-dihydroxybenzoyl)-4-O-β-D-glucopyranosyl-6-hydroxyphenylacetate | (Li et al., 2007) |
|  | Methyl 2-O-feruloyl-3-(3',4'-dihydroxyphenyl) lactate | (Liu et al., 2022) |
|  | [α-pinene](javascript:;) | (Liu et al., 2022) |
|  | β-pinene | (Liu et al., 2022) |
| Pinus Tabuliformis Carrière | [α-pinene](javascript:;) | (Liu F et al., 2024) |
|  | β-pinene | (Liu F et al., 2024) |
|  | α,α,4-Trimethyl-3-cyclohexene-1-methanol | (Liu F et al., 2024) |
|  | 3,7,11-Trimethyl-14-(1-methylethyl)-1,3,6,10-cyclotetradecatetraene | (Liu F et al., 2024) |
|  | 3-Phenyl-ethyl-2-propenoate | (Liu F et al., 2024) |
|  | [β-Citronellene](https://www.chembk.com/en/chem/(-)-BETA-CITRONELLENE) | (Liu F et al., 2024) |
